# Exploring genetic variability under extended photoperiod in lentil (*Lens Culinaris* Medik): vegetative and phenological differentiation according to genetic material’s origins

**DOI:** 10.1186/s13007-024-01135-0

**Published:** 2024-01-13

**Authors:** Mohammed Mitache, Aziz Baidani, Bouchaib Bencharki, Omar Idrissi

**Affiliations:** 1 Laboratory of Food Legumes Breeding, Regional Center of Agricultural Research of Settat, National Institute of Agricultural Research, Avenue Ennasr, BP 415, 10090 Rabat Principale, Rabat, Morocco; 2 Laboratory of Agrifood and Health, Hassan First University of Settat, Faculty of Sciences and Techniques, BP 577, 26000 Settat, Morocco

**Keywords:** Lentil, Extended photoperiod, Light, Genetic variability, Speed breeding

## Abstract

Lentil is an important pulse that contributes to global food security and the sustainability of farming systems. Hence, it is important to increase the production of this crop, especially in the context of climate changes through plant breeding aiming at the development of high-yielding and climate-smart cultivars. However, conventional plant breeding approaches are time and resources consuming. Thus, speed breeding techniques enabling rapid generation turnover could help to accelerate the development of new varieties. The application of extended photoperiod prolonging the duration of the plant’s exposure to light and shortening the duration of the dark phase is among the simplest speed breeding techniques. In this study, genetic variability response under extended photoperiod (22 h of light/2 h of dark at 25 °C) of a lentil collection of 80 landraces from diverse latitudinal origins low (0°–20°), medium (21°–40°) and high (41°–60°), was investigated. Significant genetic variations were observed between accessions, for time to flowering [40 → 120 days], time of pods set [45 → 130 days], time to maturity [64 → 150 days], harvest index [0 → 0.24], green canopy cover [0.39 → 5.62], seedling vigor [2 → 5], vegetative stage length [40 → 120 days], reproduction stage length [3 → 13 days], and seed filing stage length [6 → 25 days]. Overall, the accessions from Low latitudinal origin demonstrated a favorable response to the extended photoperiod application with almost all accessions flowered, while 18% and 57% of accessions originating from medium and high latitudinal areas, respectively, did not successfully reach the flowering stage. These results enhanced our understanding lentil responses to photoperiodism under controlled conditions and are expected to play important roles in speed breeding based on the application of the described protocol for lentil breeding programs in terms of choosing appropriate initial treatments such as vernalization depending on the origin of accession.

## Introduction

Lentil (*Lens culinaris* Medik), is one of the diploid annual legumes (2n = 2x = 14); their grains are known for their richness in proteins, minerals (Fe, K, Zn, P) and fibers [1]. Lentil grains are an important component of the daily diet for large populations in North Africa, sub-Saharan Africa, the Middle East, and the Indian sub-continent [2]. The regular consumption of lentil could help in overcoming mineral deficit for more than half of the world's population [3–5]. Lentil crop residues could also be used as livestock feed [6, 7].

Moreover lentil is a nitrogen-fixing legume that contributes to enhance soil fertility and promotes the sustainability of agricultural systems [8, 9]. The world production of lentil was in average 6.315 million tons between 2017 and 2021, with distribution according to the five continents: Asia (42%), America (42%), Oceania (10%), Africa (3%), and Europe (3%) [10].

The domestication of lentil began around 7000 B.C**.** [11] in the Near East via wild populations of *Lens orientalis* that were found in the mountains between Syria and Turkey [12–14]. After domestication, lentil with other important basic crops such as pea, faba bean, chickpea, wheat, and barley have been diffused from Near East to Greece, Central Europe, Egypt, Central Asia, and India. It has arrived in Morocco from Central Europe via Mediterranean Islands at ninth century [12, 15]. While Canada and USA started growing lentil only since 1969 and 1916, respectively [16] During domestication, several characteristics were targeted especially seed dormancy and pod indehiscence [17]. Lentil has traditionally been cultivated in Morocco, using mostly local varieties selected by farmers on the basis of a number of quality, yield, adaptation and other desired characteristics [18]. In Morocco, lentil is currently grown as a rainfed crop, in rotation with cereals. The average cultivated area is around 40,000 ha, yielding an average production of 28,163 to 41,602 tons from 2017 to 2021. [10].

The human population is expected to grow to 10 billion by 2050**,** which will put a strain on the world's resources [19]. Climate change and the emergence of new diseases and parasites threaten the productivity of agriculture worldwide [20–22]. To meet the needs of this growing population, breeders are investigating efficient methods for developing new cultivars with genetic resistance to diseases and to different abiotic stresses. However, the conventional breeding approaches adopted for enhancing the productivity of lentil take a long time and requires many years to release new and adaptive cultivars.

The extended photoperiod is one of the methods that reduce the duration of the plant cycles [23, 24]. In many studies, it has been demonstrated that extended photoperiod have a positive benefit effect for breeding, by accelerating flowering and reducing plant life cycle in Safflower [25], Strawberry [26], Soybean [27], Barley [28], Wheat [29], Chickpea [30], Faba-Bean [31] and Lentil [23, 32, 33]. The extended photoperiod can be achieved with artificial light [34]. In lentil, it has been reported that the reduction of time to flowering is favored by a photoperiod with a light intensity that can be varied around 500 μmol m^−2^ s^−1^ [35], with a duration of 16, 18, 20 and 22 h of light and 8, 6, 4 and 2 h of dark, respectively, [23, 24, 36, 37]**.** The lentil is a plant with long or neutral days [38–40]. For breeding programs based on conventional methods, in normal environmental conditions greenhouses and field conditions, the development of homozygous lines from segregating populations after hybridization takes 7–9 years, if only one generation is produced per year, while a prolonged photoperiod with continuous illumination can reduce time to flowering and accelerate growth resulting in a shorter life cycle [23].

The selection of genotypes with early flowering, early development, and high yield are among the challenges of the breeders, to adapt the crops life cycle to available growing season [41, 42]. The major element for the duration of the crop life cycle is the period between sowing and flowering, this period is regulated by the effect of temperature, photoperiod, genotype, and interactions between these parameters [43]. The temperature influence the expression of the transcription factor which directly affects the floral induction [44]. In Soybean, the light environment in which this plant is cultivated significantly influences the genotype, making it the most crucial factor [45].

To our knowledge, no studies have been done so far on the evaluation of genetic variability to the response of extended photoperiod using lentil genotypes of different latitudinal origins. The objectives of our study were (1) to analyze the genetic diversity of 80 landraces from three latitudinal origins (low, medium and high latitudes) from different countries (Russia, Serbia, Ukraine, Montenegro, Belgium, Armenia, Chile, Ethiopia, India, Iran, Afghanistan, Morocco, Italy, Turkey, and Greece) in response to the application of an extended photoperiod regime, and (2) to evaluate the sensitivity to photoperiod and select accessions that could be more adapted to use under extended photoperiod in order to use them as parents for rapid generation turnover in speed breeding growth chambers.

## Material and methods

### Plant material

**Fig. 1:**
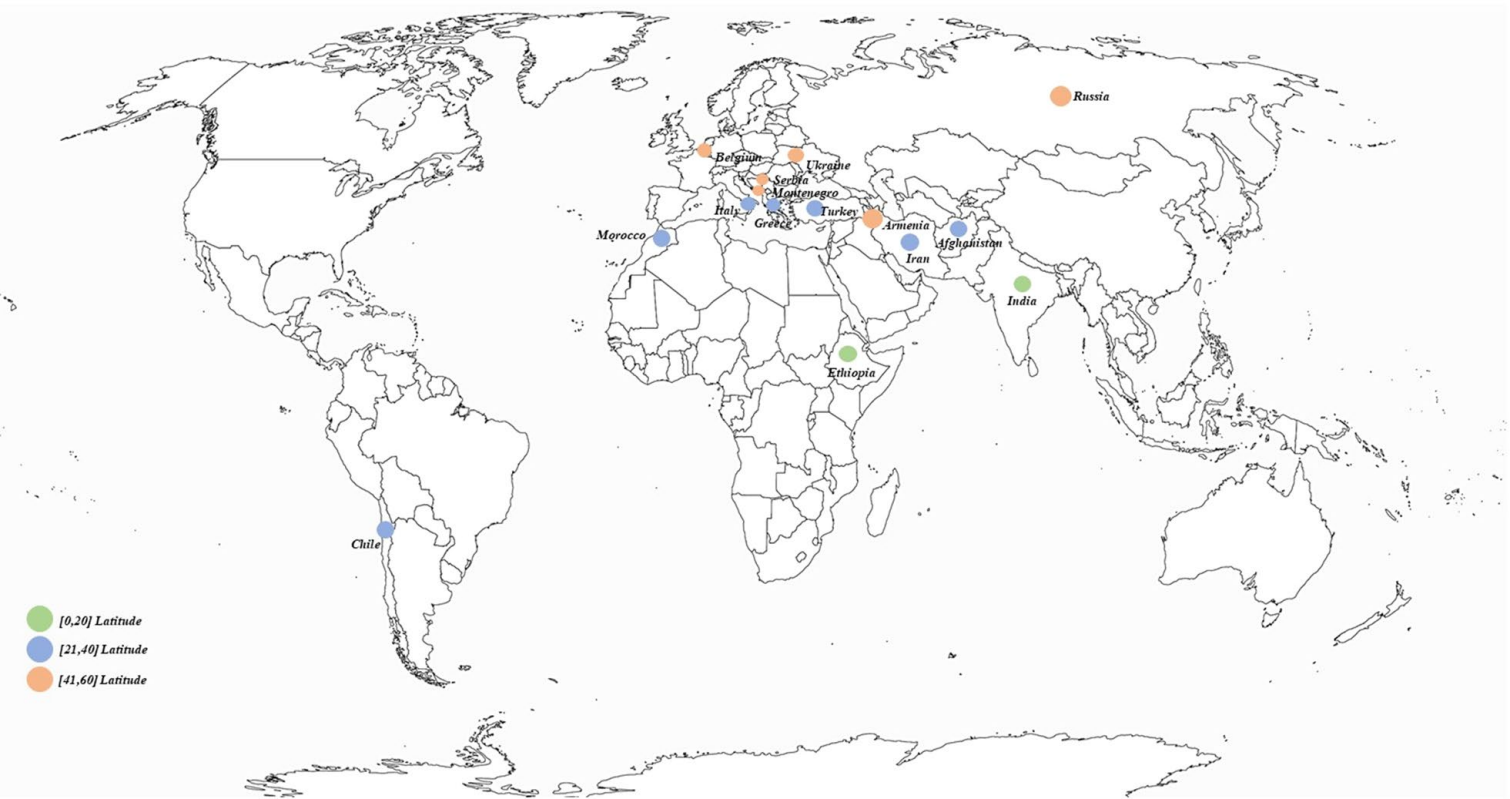
Distribution of the studied lentil accessions according to countries, latitude and longitude

A total of 80 lentil (*Lens Culinaris* Medik.) accessions from different countries (Afghanistan [3], Armenia [1], Belgium [5], Chile [13], Ethiopia [3], Greece [3], India [1], Iran [4], Italy [6], Morocco [27], Montenegro [2], Russia [3], Serbia [2], Turkey [6], Ukraine [1]) were characterized under extended photoperiod. The accessions were classified into three latitudinal origins: Low (0°–20°), Medium (21°–40°) and High (41°–60°) (Fig. 1; Table 1). The low, medium and high categories refer to the latitude 0 in reference to the equator, and therefore to the natural flowering photoperiod in the original latitude. All the accessions used in this study come from our gene bank based at the National Institute of Agricultural Research in Settat, Morocco.

**Table 1:** Classification of lentil accessions

Level	Country	Usual cropping season	Flowering time (month)	Natural daylength during flowering in country of origin (h)	Number of accessions	Latitude
Low	Ethiopia	Rainy	November–December	12–13	3	9°
	India	Summer & Rainy	August	12–14	1	20°
Medium	Chile	Spring	December-January	13–14	13	30°
	Morocco	Autumn	April–May	13–14	27	32°
	Iran	Autumn	March	13–14	4	32°
	Afghanistan	Autumn	February	13–14	3	33°
	Greece	Autumn	February	12–14	3	39°
	Turkey	Autumn	April	12–14	6	39°
	Italy	Autumn	April–May	12–14	6	39°
High	Armenia	Spring	June	14–15	1	40°
	Montenegro	Spring	May	14–15	2	42°
	Serbia	Spring	May	14–15	2	44°
	Ukraine	Spring	June	15–16	1	49°
	Belgium	Spring	May	16–17	5	51°
	Russia	Spring	June	17–18	3	60°

### Photo-thermal regime and plant growth conditions

**Fig. 2:**
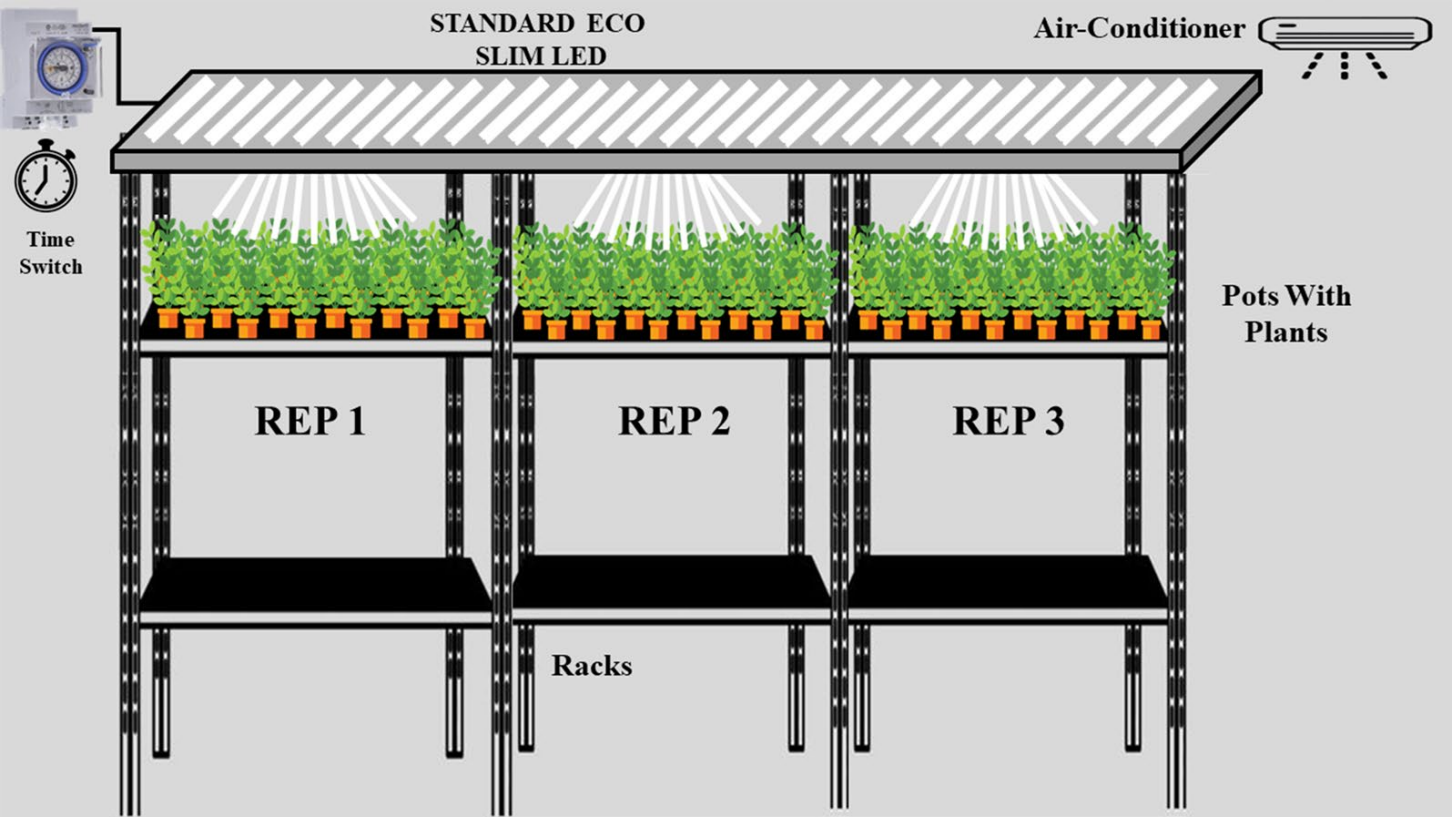
Speed breeding growth chamber

The experiment was carried out in a growth chamber at the Laboratory of Food legumes breeding, regional center of Settat, the national institute for agricultural research (INRA Morocco), during 150 days, from seed germination to plant physiological maturity. A completely randomized block design was used, with each variety planted three times. In all, three separate planting sessions were carried out, with three plants per pot for each accession, all subjected to a prolonged photoperiod treatment of 22 h of light at 25 °C and 2 h of darkness at 25 °C. Using light emitting diode (LED) Lamps (Standard ECO SLIM LED) (36 lamps of 9 W which each lamp about 14.81–18.51 µmol m^−2^ s^−1^ of light intensity) (Fig. 2).

The accessions were planted in plastic pots (500 ml capacity) filled with 2/3 of soil and 1/3 of peat compost. During the experiment, plants were irrigated every 4–7 days depending on the growth stage of the crop and the corresponding water consumption. Plants were harvested at physiological maturity. Then qualitative and quantitative measurements were made.

### Early vegetative growth, development stages and phenological characterization

Percentage of green canopy cover (GCC) corresponding to the proportion of the ground covered by plants was measured using a digital application (Canopeo) which is a mobile application, using images and video, it is an automatic color threshold image analysis tool that uses color values to classify all pixels in the image. The pixel analysis is based on blue to green (B/G) and red to green (R/G) [46]. Seedling vigor (SV) was estimated according to a scale modified [47] for the photoperiodic stress from 1 to 5, respectively (1 very poor, 2 poor, 3 medium, 4 good, 5 very good). Time to flowering (TF) Measured by counting the days of plant sowing to the day of the first flower's appearance. Time of pods set (TPS) Measured by counting the days from sowing to appearance of the first pod. Time to maturity (TM) Measured by counting the days from sowing to the yellowing and desiccation of the plant and the pods. Harvest Index (HI) was calculated according to the following formula: “Harvest index = Grain yield/Biological yield”, grain yield is a number and weight of seeds in (g), and biological yield is a dry weight of the aerial part measured after drying in an oven at 70 °C for 48 h. The vegetative stage length (VGS) corresponds to the number of days after sowing until the appearance of the first flower. While the reproductive stage length (RPS), corresponds to the number of days from the appearance of the first flower to the formation of the first pod. Finally, the seed filling stage length (SFS), corresponds to the number of days from the appearance of the first pod until 80% maturity.

### Statistical analysis

For each parameter, descriptive statistics, analysis of variance were performed to test the effect of genotype and latitude under speed breeding by extended photoperiod. In addition, in order to assess the hypothesis of differentiation of the accessions according to their geographic origins in response to the application of the extended photoperiod and to determine the contribution of each trait in discriminating between origins, a canonical discriminant analysis was carried out by using the Statistical Package for the Social Sciences (SPSS) database software, version 21 for Windows. Graphical extrapolation of the results was performed using Microsoft Excel and (SPSS). While R software was used for variance analysis and through the "*agricolae*" package [48]. Duncan post-hoc test was used to test the differences between the different light intensity treatments by the “*multcomp*” R package [49]. The principal component analysis was performed using the R package ‘*FactoMineR*, *factoextra’* [50].

## Results

### Genetic variation of vegetative and phenological traits

The analysis of variance revealed a significant effect (p ≤ 0.05) of genotype on all traits, and a significant variation was also observed according to the latitudinal origins for all traits except reproduction stage and seed filling stage length (Table 2).

**Table 2:** Mean squares from ANOVA results of quantitative and qualitative traits of lentil as influenced by photoperiodic regime

SOV	Df	GCC (%)	HI	SV	TF (days)	TPS (days)	VGS (days)	RPS (days)	SFS (days)	TM (days)
Genotype	79	2.7867***	0.0089***	0.6784*	229.83***	206.59***	229.83***	5.996*	22.35	91.44**
Latitude	2	25.164***	0.0531***	1.878*	809**	280	809**	1.592	10.11	221.92*

Signif. codes: 0.000 ‘***’ 0.001 ‘**’ 0.01 ‘*’ 0.05 ‘.’.

*SOV* source of variation, *TF* time to flowering, *TPS* time of pods set, *TM* time to maturity, *HI* harvest index, *GCC* green canopy cover, *SV* seedling vigor, *VGS* vegetative stage length, *RPS* reproduction stage length, *SFS* seed filing stage length

### Genetic variation of accessions from the latitudinal origins

**Fig. 3:**
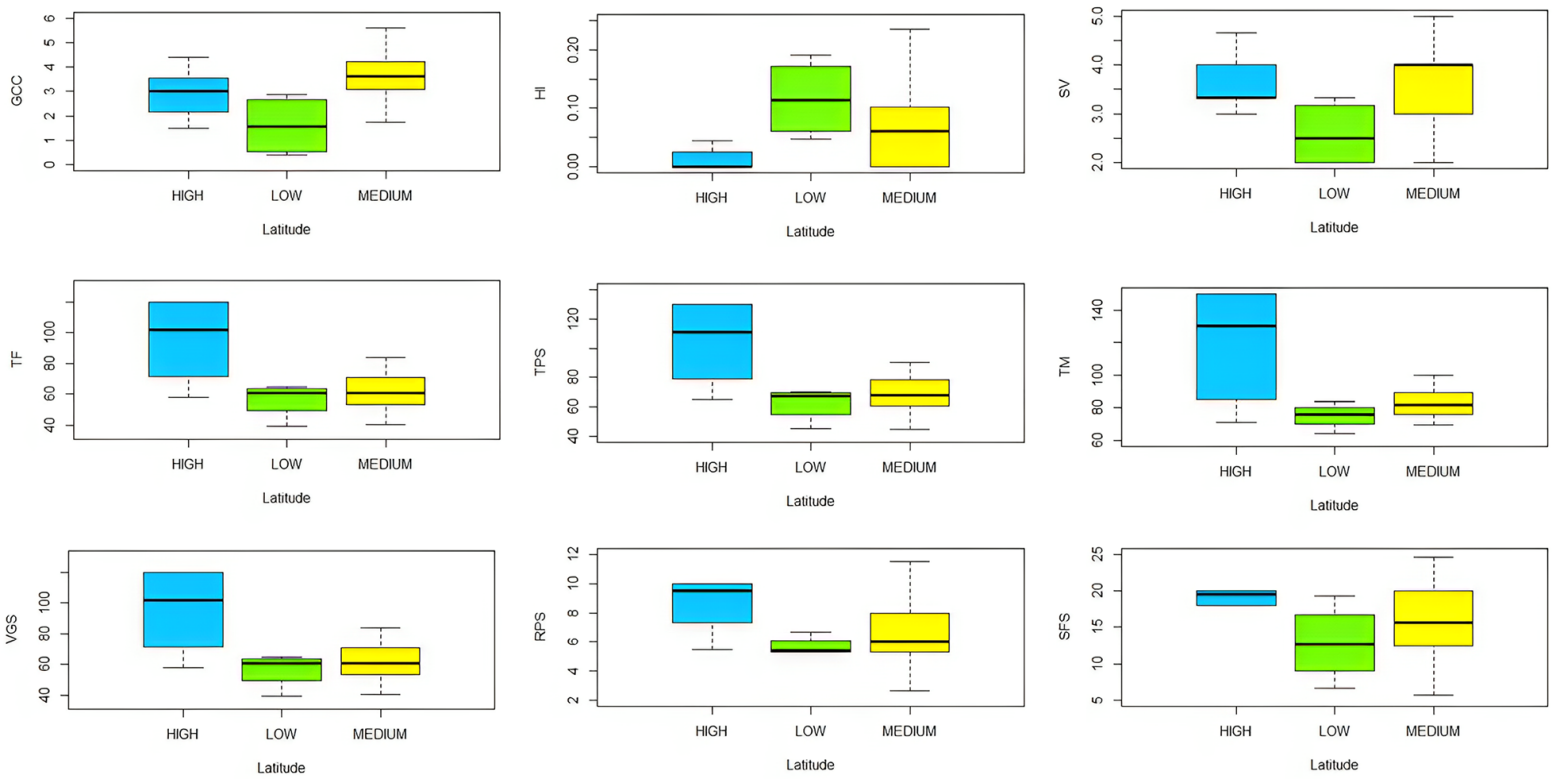
Boxplot of comparison between lentil origins under the extended photoperiod conditions. *TF* time to flowering, *TPS* time of pods set, *TM* time to maturity, *HI* harvest index, *GCC* green canopy cover, *SV* seedling vigor, *VGS* vegetative stage, *RPS* reproduction stage, *SFS* seed filing stage

In this study, an analysis of genetic variation was carried out on accessions from the three distinct latitudinal origins: low, medium and high latitudes. To visualize and compare this genetic variation, we used boxplots (Fig. 3). Through observation of these boxplots, we identified interesting trends in genetic variation between latitudinal origins. Important differences in genetic distributions are clearly discernible between the Low, Medium and High latitudinal origins (Fig. 3; Tables 2, 3). These results suggest a distinct genetic structuring between different latitudinal groups, highlighting the potential influence of environmental and latitudinal factors on the genetic diversity of the studied accessions.

**Table 3:** Effect of photoperiod on traits for different lentil origins

Traits latitude		HI	GCC (%)	SV	TF (days)	TPS (days)	TM (days)	VGS (days)	RPS (days)	SFS (days)
High	0.011a		2.91a	3.43a	96.04a	104.71a	121.43a	96.04a	8.67a	16.71a
Medium	0.063b		3.55a	3.66a	68.88b	75.7b	91.51b	68.88b	6.83ab	15.81a
Low	0.12c		1.6b	2.58b	56.54b	62.25b	75.08b	56.54b	5.71b	12.83a

The table values represent (Means), “a, b and c” Duncan test

*TF* time to flowering, *TPS* time of pods set, *TM* time to maturity, *HI* harvest index, *GCC* green canopy cover, *SV* seedling vigor, *VGS* vegetative stage length, *RPS* reproduction stage length, *SFS* seed filing stage length

These results boost our understanding of genetic diversity in a geographical context, which could have important implications for the preservation and future use of these genetic resources in crop improvement and biodiversity conservation programs.

### Genetic variability of vegetative growth under extended photoperiod

Vegetative cover rate showed significant differences for the 80 accessions, those from low latitudinal origin showed a low percentage of vegetative cover with 1.6%. While, those from Medium latitudinal origin showed a higher percentage of vegetative cover with 3.66%, same trend was observed for seedling vigor (Table 3). For vegetative cover and seedling vigor, two distinguished groups were observed according to Duncan test (Table 3).

### Genetic variability of phenological stages under extended photoperiod

Significant differences obtained according to Duncan test among the studied accessions were observed for phenological stages between Low, Medium and High latitudes (Table 3). For the flowering time, the Low latitudinal accessions were the earliest ones at flowering with an average of 69 days after sowing, while the accessions from the High latitude were the latest at flowering with an average of 96 days after sowing (Table 3). For the time of pods set, the Low latitudinal accessions, have averages of 62 days after sowing being the earliest, in contrast to the accessions from High latitude as the latest, with averages of 104 days after sowing (Table 3). Time to maturity registered a difference between the accessions, low latitudinal accessions, in average with 75 days after sowing as the earliest, whereas the accessions from the High latitude as the latest in average with 121 days after sowing. Regarding vegetative, reproductive and seed filling stages, the Low latitude accessions have the lowest number of days values.

**Fig. 4:**
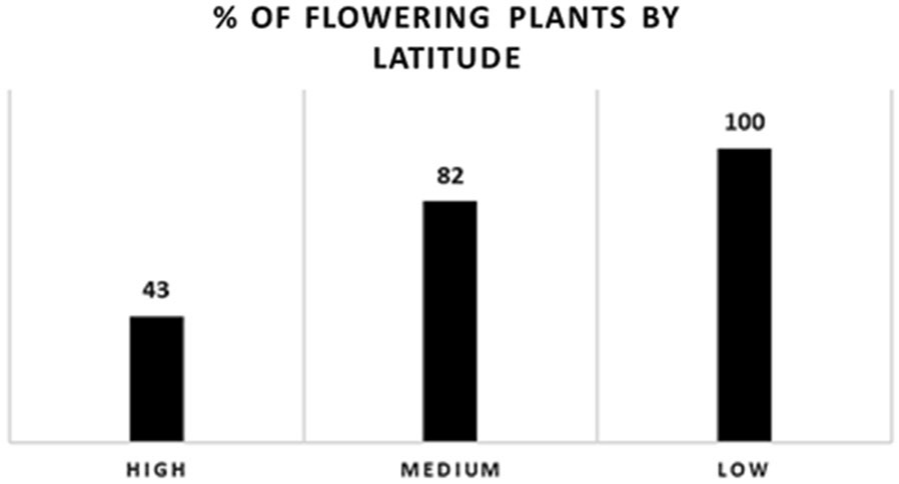
Variation in flowering percentage between different latitudinal origins of lentil accessions

It should be noted that some accessions of Medium latitudinal origin (18%) and High latitudinal origin (57%) failed to flower, while all accessions from Low latitudinal origin have flowered (Fig. 4).

### Variation of harvest index according to genotype under extended photoperiod

The yield and biomass measurements allowed us to estimate the harvest index for the different accessions with a very highly difference, the accessions from the High latitude presented the lowest index with 0.011, while the accessions from Low latitude presented the highest index with 0.12, and three distinguished groups are showed by using Duncan test (Table 3).

### Correlation between different traits

**Fig. 5:**
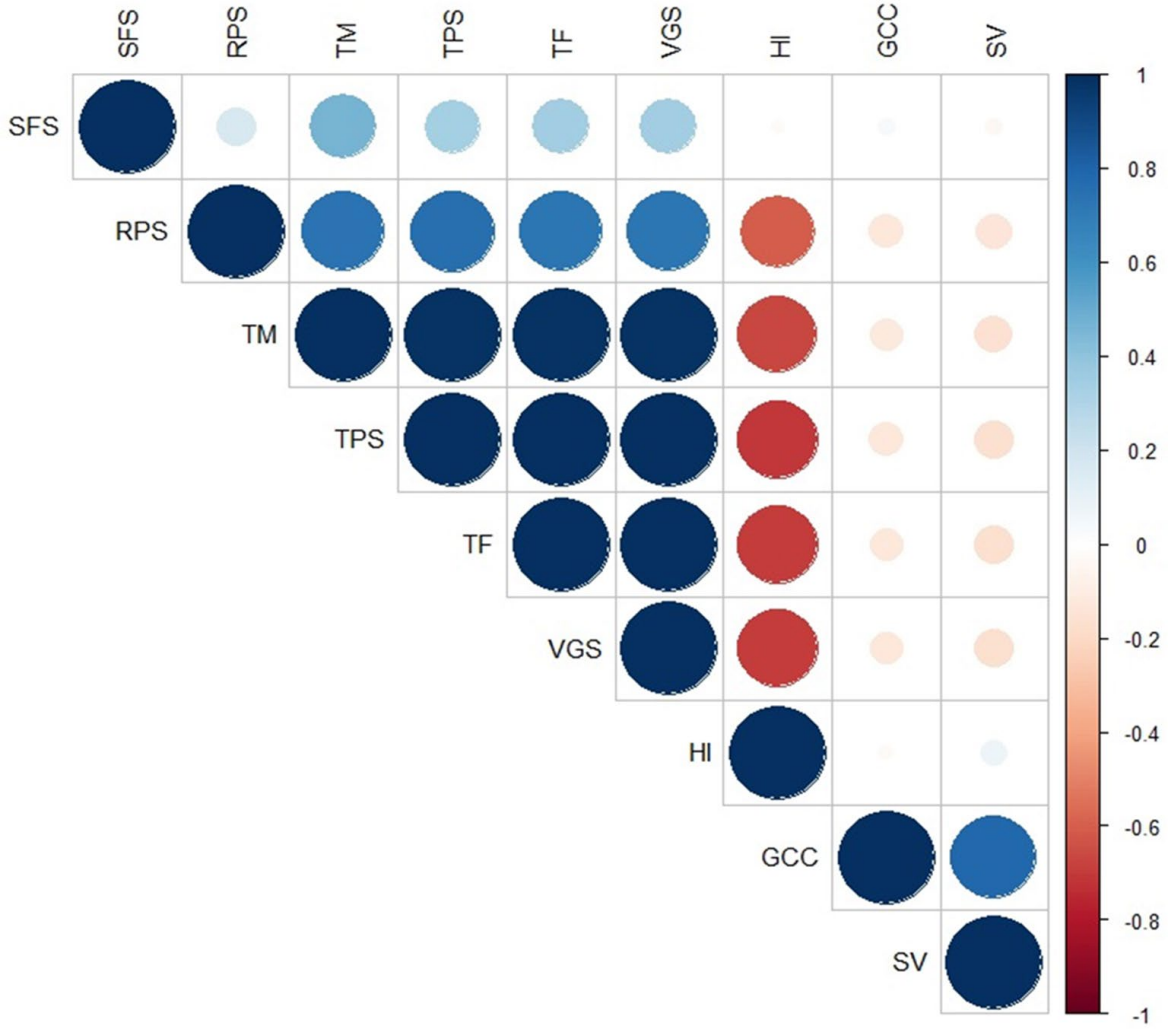
Pearson correlation matrix of variables under extended photoperiod. *TF* time to flowering, *TPS* time of pods set, *TM* time to maturity, *HI* harvest index, *GCC* green canopy cover, *SV* seedling vigor, *VGS* vegetative stage length, *RPS* reproduction stage length, *SFS* seed filing stage length

The Pearson correlation method was used to examine the links between the variables in our study (Fig. 5).

In our study, we observed a Pearson correlation coefficient of 0.79 between the GCC and SV variables, indicating a strong positive correlation between them. This suggests that an increase in vigor is generally associated with an increase in canopy cover and vice versa, in the same trend the phenological traits showed strong positive correlations with each other TF and VGS with (1.00), TF and RPS with (0.72), TF and TPS with (1.00), TF and TM with (0.99). In contrast, the variables HI against TF, VGS and TPS had a correlation coefficient of (-0.70), indicating a negative correlation between them, and this suggests that a prolongation of phenological stages has a negative influence on yield under extended photoperiod.

### ***Multivariate analysis***

#### Canonical discriminant analysis

To test the hypothesis of differentiation of the studied accessions according to their latitudinal origins, a canonical discriminant analysis was carried out, providing a graphical view that illustrated the existence of groups using the origin of accessions (Low, Medium and High latitudes) as a dependent variable. Time to flowering, time of pods set, time to maturity, harvest index, green canopy cover, seedling vigor, vegetative stage length, reproduction stage length and seed filing stage length as an explicative variables. The two first functions were significant, for the first function, Wilks' Lambda (0.57), Chi-square (41.48) and P < 0.000, and for the first function, Wilks' Lambda (0.76), Chi-square (20.44) and P < 0.001. The first function explained 50.8% while the second function explained 49.2% of the total variance, and corresponding to the correlations of 0.5 and 0.49, respectively.

**Fig. 6:**
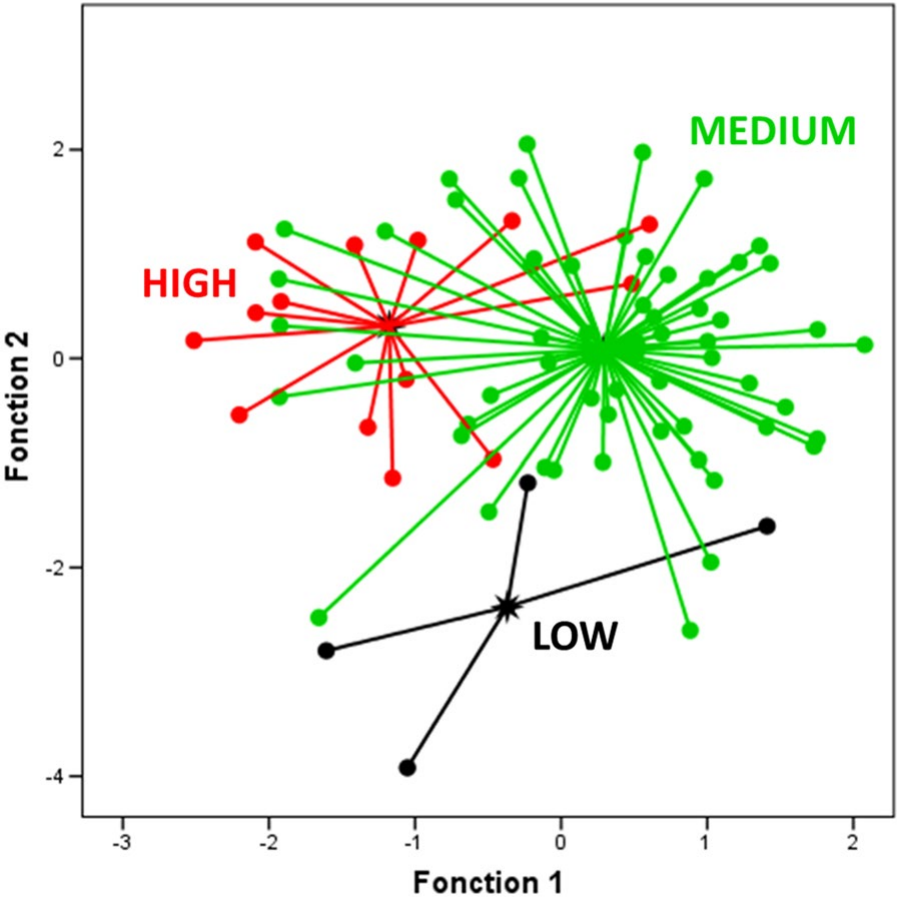
2D scatter plot showing the distribution of accessions according to the two discriminant functions obtained by DFA for the studied traits under extended photoperiod conditions

The two-dimensional (2D) Scatter diagram of the discriminant space (canonical plot) (Fig. 6) presented the distribution of samples separated by the first two functions. Based on the standardized coefficients of the Discriminant Function Analysis (DFA), the accessions from High latitudinal origin were highly weighted in the negative part of DFA-F1, while those from Medium latitudinal origin are the most weighted in the positive part of DFA-F1. The accessions from Low latitudinal origin was clearly distinguished from the other origins by the function 2.

These results suggest that discriminant analysis has successfully identified distinct characteristics between the groups, enabling them to be effectively discriminated in this reduced two-dimensional space.

#### Principal component analysis

**Fig. 7:**
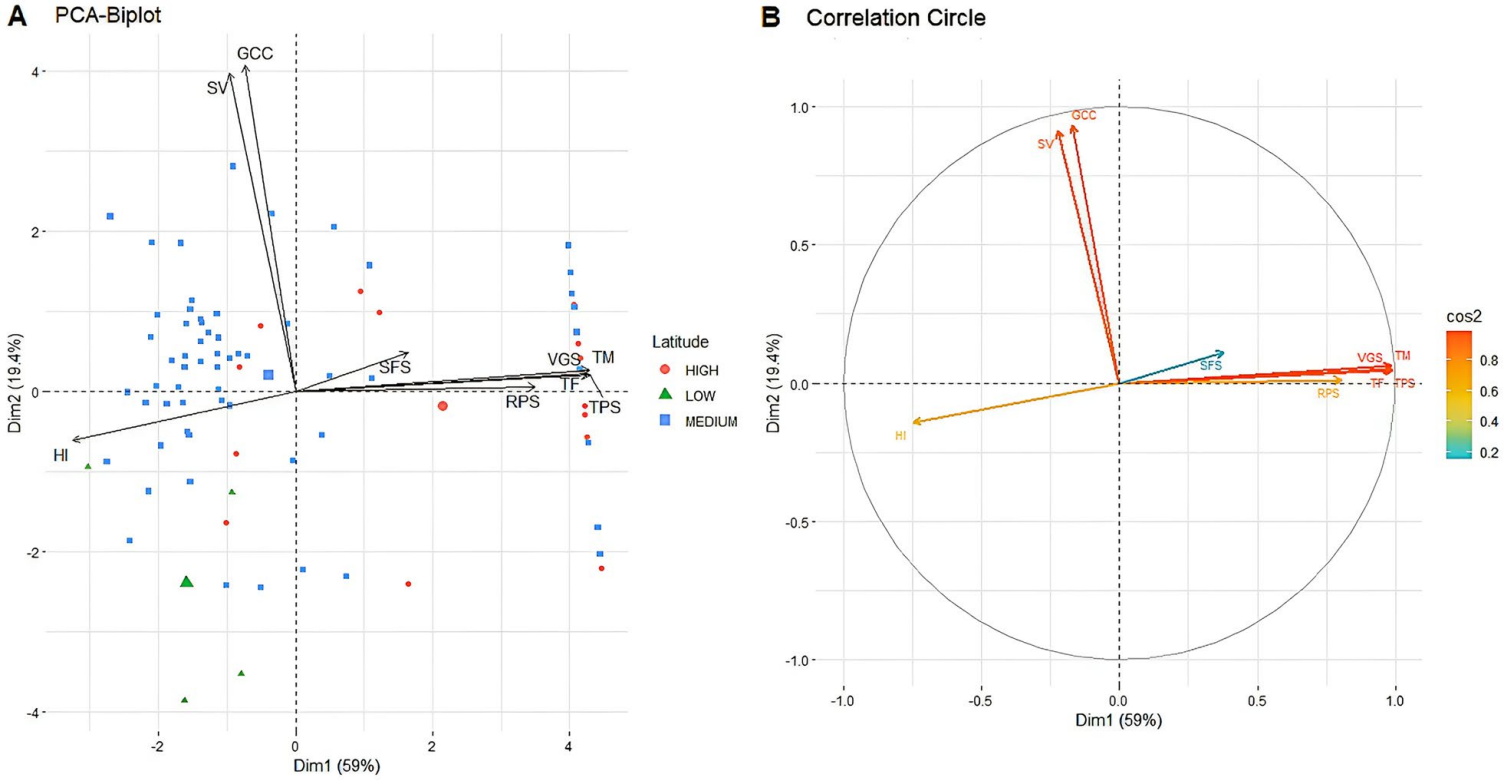
Principal component analysis of lentil traits and origins under extended photoperiod. **a** PCA-Biplot, **b** Correlation circle. TF time to flowering, *TPS* time of pods set, *TM* time to maturity, *HI* harvest index, *GCC* green canopy cover, *SV* seedling vigor, *VGS* vegetative stage length, *RPS* reproduction stage length, *SFS* seed filing stage length

Principal component analysis was applied based on the mean values of all variables for the extended photoperiod treatment. The first two components explained 59% for the first axis PCA1, 19.4% for the second axis PCA2 (Fig. 7A). The first principal component was highly and positively correlated with time to flowering (0.98), vegetative stage length (0.98), time of pod set (0.99), and time of maturity with (0.98), while highly and negatively correlated with harvest index (− 0.75). The second principal component was strongly and positively correlated with green canopy cover (0.93) and with seedling vigor (0.91).

## Discussion

Many studies on lentil have focused on the effect of genotype on physiological and morphological traits under normal temperature and photoperiod conditions in either the controlled environments or field. However, studies on the sensitivity to prolonged photoperiod are limited. Therefore, our study aims to investigate the effect of genotype and latitudinal origin of different lentil accessions in an extended photoperiod environment.

### Implications of latitudinal origin on photoperiodic response

The impact of light conditions on flowering and plant development is of great importance, particularly in the context of plant adaptation to varying climates and daylengths. Plants are sensitive to the duration of daylight and darkness, which influences the start of flowering [51]. Long or short photoperiods can modify the flowering period according to the specific needs of each species or cultivar. For instance, rice plants from equatorial regions prefer shorter days to start flowering, while those from regions further from the equator require longer days [52]. Moreover, plants have the ability to adjust to environmental variations, including changes in photoperiod. When transplanted to new environments, plants can recalibrate their internal circadian clocks to adjust to local photoperiods [53]. Plants have photoreceptors, such as phytochromes and cryptochromes, that enable them to detect light, including red (R) and blue (B) light [54]. When activated by light, these photoreceptors initiate specific signaling pathways, acting as molecular switches. Light signals are integrated into the plant circadian clock, composed of genes and proteins that regulate gene expression throughout the day, among these genes FLOWERING LOCUS T (FT) plays a key role in regulating flowering in response to specific light signals [55].

The diverse latitudinal origins of the lentil accessions in our study could potentially contribute to the variations observed in their photoperiodic responses. Geographic latitude, longitude and climate may influence the natural photoperiod to which these accessions have adapted over generations [27, 56]. This adaptation could have caused variations in their sensitivity to extended photoperiods, as proven by [57] that some wild lentil genotypes are less sensitive than cultivated ones to light quality. The response to photoperiodism is a major factor in determining the timing of flowering, and is governed by the complex interplay between internal circadian rhythm and external day length, which varies according to geographical latitude [27].

### Impact of extended photoperiod on genetic variation of phenological and reproductive stages

The various lentil genotypes studied comes from different geographical origins and have distinct genetic characteristics, resulting in varying sensitivity to environmental factors such as extended photoperiod. Previous research [32] shows that some genotypes from different origins can show increased or reduced sensitivity to specific environmental conditions such as long days, vernalization and temperature. The application of extended light duration induced an early flowering of the long day plants (LDP) such as lentil and chickpea [58]. Similar results have been reported by [23] on advanced lines, local populations, and wild accessions (*Lens orientalis*) in lentil. According to [31], the time from sowing to flowering differed significantly among accessions and also varied with the photoperiodic regimes. Moreover, [48] studied the responses of cultivated and wild-type lentil accessions in a growth chamber under controlled conditions (22◦C/16 h during the day and 16◦C/8 h at night) in different light environments (red/far-red ratio (R/FR) and photosynthetically active radiation (PAR)), the authors showed that time to flowering were significantly influenced by genotype, light environment, and the interaction between them, and this is related to the origin of each accession. In the present study, several lentil accessions showed a significant delay in their flowering process, or even failed to flower within the experiment's period. The reason for this response resides mainly in the photoperiod, i.e. the duration of day and night light to which these plants are subjected. The accessions from higher latitudes, such as Russia, showed a marked tendency to delay flowering by more than 80 days, and 57% of these plants failed to flower (Fig. 4), in this experiment, even under a prolonged photoperiod. This is explained by their genetic adaptation to environments where flowering days are characterized by a notably prolonged photoperiod, with up to 17 h of natural light as shown in the Table 1. Therefore, they may need to be given a longer light duration than their natural light duration, more than 22 h of light or continuous light (24 h of light) in a period between plant emergence to flowering, to become sensitive to this light duration and accelerate their flowering process as LDP [59, 60]. Another hypothesis is that there is a possible need for vernalization for this group of accessions, a process where prolonged exposure of lentil seeds to cold temperatures may be necessary to induce and accelerate flowering [32]. If the genetic variability observed in response to photoperiodism is associated with the potential role of vernalization, it may highlight the complex interaction between genetic and environmental factors in the regulation of flowering. Further research questions, that needs future exploration, related to the response of these accessions and their progenies (after crosses) to extended photoperiod under speed breeding in terms of flowering arise. In contrast, accessions from Ethiopia and India, located at lower latitudes, have evolved naturally to prosper in more balanced light conditions, with days and nights typically lasting 12 to 14 h during their flowering period, as shown in the Table 1. Previous studies of lentil response to photoperiod have shown that genotypes from subtropical regions were less sensitive to variations in daylength [61]. These observations highlight the essential impact of plants' genetic adaptation to their local environment, particularly with regard to the length of day and night, knowledge that is crucial to breeding and selection programs aimed at developing varieties adapted to specific regions. Therefore, the influence of photoperiod on plant growth and development is mainly linked to the regulation of the long-day-dependent flowering pathway, such as the FLOWERING LOCUS T (FT) pathway [55]. Instead of directly accelerating photosynthesis, a prolonged photoperiod promotes the transition to the early flowering phase by modulating this signaling pathway.

Significant genetic variability was observed for the duration of different development stages. The extended photoperiod significantly influenced the development stages of each genotype as proved by [62]. Although vegetative stage length and reproductive stage length were positively correlated with the genotypes earliness, except seed filling stage revealed the opposite, this is explained by the time compensation for the duration of the vegetative phase which will determine the rate and duration of seed filling stage length as proved by [63].

### Effect of extended photoperiod on genetic variability of vegetative growth

In our study, a high genetic variability (p = 0.000) was observed for green canopy cover between the studied accessions. Canopy cover, determined by the Canopeo, the Green canopy cover, is a very important parameter for biomass estimation based on the percentage of green color of plants [64]. However, the photoperiod regime can influence the growth and development of the plant as proven by [24]. Significant genetic diversity was observed among accessions with regard to seedling vigor. This trend has also been observed in lentil plants exposed to drought stress and well-watered conditions in previous studies [47, 65], and it means that when plants are subjected to stress conditions, whether controlled or not, they grow differently, tolerating the stress or being sensitive to it.

The importance of our study coincides with current efforts in the field of speed breeding and genetic improvement. The significant genetic variability observed for key traits such as flowering time, developmental stages and harvest index under extended photoperiod conditions has significant implications for accelerated breeding. As breeders and researchers work to develop crop varieties with improved performance and yield potential, it is crucial to understand the genetic basis of rapid development. Our results provide a valuable information on the possibility of exploiting genetic diversity to speed up the breeding process and obtain the desired characteristics through SB techniques. By revealing the complex interaction between genetic diversity and photoperiodic responses, our study offers a valuable gateway to the targeted manipulation of reproductive traits, enabling the rapid creation of high-yielding crop varieties.

## Conclusion

The results of this study indicated that extended photoperiod highly influenced the growth and development of different lentil genotypes. However, a large genetic variability for response to prolonged photoperiod was observed among the different accessions. Genotypes of Low latitudinal origin showed early flowering and maturity, and high yield, therefore, higher adaptability and easy use under extended photoperiod conditions without any strategies of initial induction of flowering (vernalization…). While, many other accessions especially from High and Medium latitudinal origins did not flower during this experience, therefore, using these accessions under extended photoperiod would be difficult and needs additional initial steps such as vernalization that could slow down the speed breeding process. Hence, our results suggest that Low latitudinal and some Medium latitudinal accessions are more recommended for breeding programs applying extended photoperiod to accelerate plant growth and flowering.

## Data Availability

The datasets used and/or analysed during the current study are available from the corresponding author on reasonable request.
